# Age-related cardiovascular disease and mosaic hematopoietic loss of the Y chromosome

**DOI:** 10.20517/jca.2024.29

**Published:** 2025-07-08

**Authors:** Nicholas W. Chavkin

**Affiliations:** 1 Center for Developmental Biology and Regenerative Medicine, Seattle Children’s Research Institute, Seattle, WA 98101, USA; 2 Department of Pediatrics, Division of Cardiology, University of Washington School of Medicine, Seattle, WA 98105, USA; 3 Institute for Stem Cell and Regenerative Medicine, University of Washington, Seattle, WA 98109, USA

**Keywords:** Sex chromosome aneuploidy, mosaic Loss of Y chromosome, age-related cardiovascular disease, mosaic chromosomal alterations, clonal hematopoiesis

## Abstract

Age is the strongest risk factor for cardiovascular morbidity and mortality, driven in part by aging leukocytes negatively impact cardiovascular health. This effect is particularly pronounced in men, who have a shorter average lifespan-approximately six years less than women, largely due to higher mortality rates in old age. One contributing factor is a male-specific aging blood phenotype characterized by the mosaic loss of the Y chromosome (mLOY), a condition in which a subset of blood cells lose the Y chromosome. mLOY is highly prevalent in elderly men, affecting 45% of those aged 70 and older. Recent studies have linked mLOY to increased early mortality, age-related pathologies, and cardiovascular disease, potentially explaining the observed sex discrepancy in life expectancy. Experimental studies have begun to uncover potential mechanisms related to leukocyte responses to cardiac injury and the polarization of macrophages that promote pro-fibrotic cytokine expression. Current evidence suggests that mLOY reflects an underlying aspect of biological aging related to genomic instability, which drives age-related diseases, including cardiovascular conditions. Although mLOY shares similarities with general age-related changes in the hematopoietic system, it may exert distinct effects on leukocytes that promote cardiovascular disease through enhanced tissue fibrosis pathways. These findings highlight that mLOY accumulates with age and contributes to cardiovascular disease through mechanisms that are independent of biological aging per se. Further investigation into mLOY-specific pathways in aging and age-related diseases may reveal novel therapeutic targets for a chronic condition that affects a large proportion of the elderly male population. This review discusses the current literature on mLOY and its connection to cardiovascular disease within the broader context of aging.

## Graphical Abstract

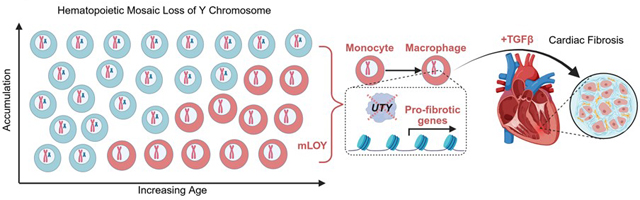

## INTRODUCTION

**Figure 1.**
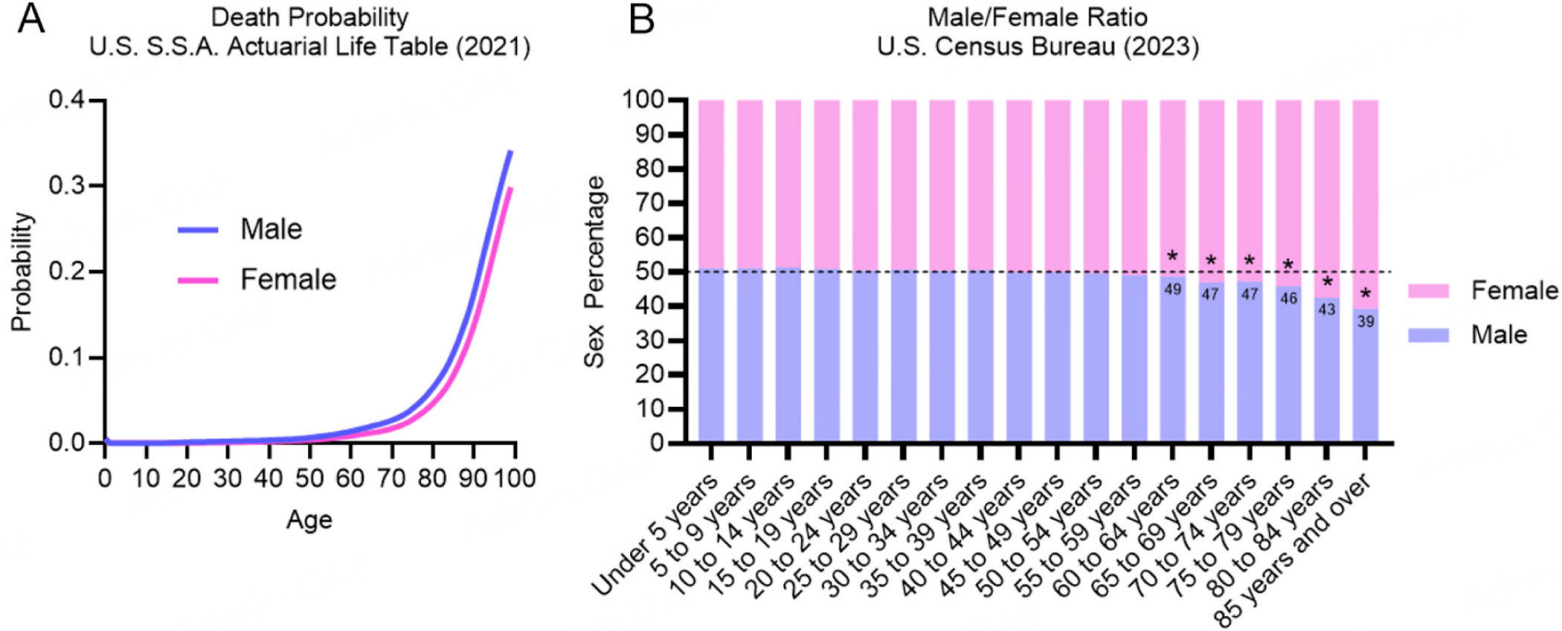
Mortality Rate and Sex Ratio by Age. (A) Death probability by age for males and females, based on the 2021 actuarial life table from the U.S. Social Security Administration (SSA). (B) Male-to-female ratio by age group, based on the 2023 U.S. Census Bureau report on population distribution. Percentages represent the proportion of males or females within each age group. Statistical analysis was performed by Chi-squared test with Bonferroni correction, comparing observed sex ratios against an expected 50:50 distribution. Asterisks (*) indicate adjusted p-values < 0.05. For statistically significant age ranges, the percentage of males is reported.

Biological sex is a predictive factor for life expectancy. It is widely established that male sex is correlated with a shorter average life expectancy - approximately six years less - in developed areas around the world (USA: 79 years for females *vs*. 73 years for males; Europe: 83 *vs*. 77; Japan: 88 *vs*. 82, based on currently available national census data). Interestingly, population and mortality data from the United States suggest that this discrepancy becomes apparent in older age. Men exhibit a noticeably higher mortality risk than women starting around 60 years of age. As a result, the male-to-female sex ratio in elderly people decreases significantly from age 60 onward [Figure 1]. According to the New England Centenarian Study, only 15% of individuals who live to 100 years old are men, and just 10% of those reaching 110 years old are male^[1]^. These figures suggest that biological aging may have a greater impact on men than on women.

Understanding sex-related differences in aging from a biological perspective is key to uncovering why men seem to age faster than women. Moreover, mechanistic insights into these differences can help identify key drivers of aging and age-related diseases. Recent studies have defined sex-specific aspects of aging that affect leukocytes and contribute to age-related diseases. It is now understood that leukocytes can tolerate the loss of one sex chromosome, resulting in hematopoietic mosaic loss of the Y chromosome (mLOY) in men or hematopoietic mosaic loss of the X chromosome (mLOX) in women. Although such mosaic chromosomal alterations were historically difficult to study, recent advances in the identification and analysis of mLOY have enabled researchers to elucidate both correlations and causal effects. Notably, mLOY has been linked to age-related cardiovascular disease, the leading cause of morbidity and mortality worldwide, underscoring its potential impact on aging phenotypes. This review summarizes recent studies on mLOY, examining its correlation with age-related cardiovascular disease and exploring its underlying mechanistic consequences. Our goal is to provide an up-to-date overview of current knowledge on mLOY and its role in cardiovascular aging.

### Genomic instability in aged leukocytes and cardiovascular disease

Age is the most significant risk factor and strongest predictor of cardiovascular disease^[2]^. Both cardiovascular morbidity and mortality increase exponentially with age, and advancing age amplifies the incidence of cardiovascular disease associated with modifiable risk factors^[3–5]^. Molecular changes in leukocytes that occur with aging may contribute substantially to this increased risk. Leukocyte age can be estimated in blood samples by measuring telomere shortening^[6]^. Using this approach, increased leukocyte age has been linked to a higher incidence of atherosclerosis, left ventricular diastolic dysfunction, carotid intima-media thickness, cardiometabolic conditions, elevated diastolic blood pressure, myocardial infarction, stroke, coronary artery disease, endothelial dysfunction, and chronic heart failure^[7–13]^. Molecular characterizations of aged leukocytes also include epigenetic changes. DNA methylation patterns associated with aging have been used to develop “epigenetic clocks”, which provide a more precise definition of aged leukocytes than biological age^[14]^. These epigenetic markers of leukocyte aging correlate with increased mortality risk^[15]^ and poorer cardiovascular health^[16,17]^. One study found blood-based epigenetic signatures that reflect cardiac biological age, as well as corresponding changes in cardiac tissue^[18]^. Aging also affects hematopoietic stem cell differentiation, skewing lineage potential toward a myeloid bias and reducing lymphoid cell populations^[19]^. In the UK Biobank, cardiovascular mortality correlates with shifts in leukocyte composition, notably reduced lymphocyte counts and increased monocytes and neutrophils^[20]^. Collectively, these findings support the idea that aged leukocytes contribute to cardiovascular morbidity and mortality.

Aged leukocytes are known to accumulate somatic mutations due to genomic instability, one of the hallmarks of aging. During proliferation, somatic mutations can occur at a rate that outpaces the capacity of DNA mismatch repair mechanisms^[21]^. Hematopoietic stem cells are particularly susceptible to these mutations due to their high proliferative capacity^[22]^. When these cells acquire point mutations in specific driver genes, they may gain a competitive advantage over non-mutant counterparts, leading to clonal expansion. This phenomenon is termed Clonal Hematopoiesis of Indeterminate Potential (CHIP)^[23,24]^. These mutations can be detected in blood samples using Whole-Genome or Whole-Exome Sequencing, targeted sequencing of frequently mutated genes, error-corrected sequencing, or single-cell RNA sequencing coupled with targeted analysis^[25,26]^. The prevalence of CHIP mutations increases exponentially with age and is associated with a higher incidence of age-related cardiovascular conditions such as ischemic heart failure, atherosclerosis, and venous thrombosis^[23,27–29]^. Additional risk factors for CHIP include smoking, environmental toxin exposure, genetic predisposition, obesity, and chemotherapy^[23,30]^. Mechanistic studies have shown that CHIP-associated mutations drive pro-inflammatory changes in leukocytes, enhancing leukocyte recruitment and inflammatory responses in cardiovascular diseases such as heart failure and atherosclerosis^[27]^. However, known CHIP-driver mutations account for only about 15%−20% of total clonal expansions in leukocytes^[31–33]^. Notably, CHIP driven by unidentified mechanisms is also correlated with increased mortality^[31,34]^. Recent studies have begun to shed light on these unexplained CHIP events by identifying clonal hematopoiesis arising from mosaic chromosomal alterations, large-scale DNA rearrangements or deletions that were previously difficult to detect using traditional sequencing approaches^[35,36]^. These mosaic chromosomal alterations are broadly correlated with increased mortality and many age-related diseases, including cardiovascular disease^[37–40]^. Together, these insights highlight a crucial link between genomic instability in aged leukocytes and the development of cardiovascular disease.

### Detection methods and prevalence of loss of Y chromosome

The most frequent somatic chromosomal alterations in leukocytes occur in the sex chromosomes: X and Y^[41]^. Loss of the Y chromosome was first observed in non-cancerous cells over 50 years ago. Since then, several studies have reported an age-related increase in mLOY using fluorescent imaging of chromosomes in blood cells from men, leading to the hypothesis that mLOY is a normal, age-related phenomenon in hematopoietic cells^[42–44]^. Notably, a single cell can tolerate the loss of the Y chromosome, yielding an “X0” sex chromosome configuration, without triggering catastrophic necrosis or apoptosis. Although Y chromosome gene expression is relatively low in post-somatic tissues compared to the testis, it is not negligible; several genes in the male-specific region of the Y chromosome (MSY) are differentially expressed across various tissues^[45]^. Additionally, many MSY genes have homologous counterparts on the X chromosome that share high sequence homology and functional redundancy, with similar expression patterns^[45]^. Most of these homologous X-linked genes escape X-chromosome inactivation in female cells^[46]^. Therefore, Y chromosome loss may be tolerated in part due to the relatively low expression of MSY genes and the resulting partial haploinsufficiency, rather than a complete loss of function. Correlating mLOY with clinical outcomes through quantification of sex chromosome aneuploidy has long been challenging, but recent advances in detection methods have significantly improved the feasibility and accuracy of such analyses.

Recent methods for mLOY detection and quantification in blood leverage next-generation sequencing and bioinformatic or molecular approaches. These methods build upon previous techniques, such as Fluorescence *in situ* Hybridization (FISH), which allowed the identification of individual cells lacking the Y chromosome in blood smears and enabled quantification of mLOY in patients^[47]^, to now support analysis in much larger cohorts. Whole-genome sequencing (WGS) and whole-exome sequencing (WES) are routinely applied to blood samples from large population-based cohorts, providing both Single Nucleotide Polymorphisms (SNPs) data and general read coverage of the X and Y chromosomes. These data can be used to estimate the extent of mLOY and mLOX in each individual^[48–51]^. Two main computational methods have been developed for this purpose. The first involves analyzing SNPs in the non-pseudoautosomal region (non-PAR) of the Y chromosome. A reduction in expected signal intensity in this region can be used to infer mLOY through a method termed “mLRR-Y”^[50,52–54]^. A second, more recent approach - termed “PAR-LOY” - analyzes the pseudoautosomal region (PAR) of the Y chromosome. This method compares allele frequencies in the PAR of the Y chromosome to those of the homologous PAR on the X chromosome to identify mosaic chromosomal alterations in the Y chromosome^[48]^. Additionally, mosaic chromosomal alterations across all chromosomes, including the Y chromosome, can be detected in WGS/WES data using dedicated bioinformatic tools, such as a mosaic chromosomal alteration caller^[35]^. The Y chromosomal-specific output from such tools can also be used to determine mLOY^[55]^, often with a sensitivity similar to that of PAR-LOY. More direct quantification of mLOY can be achieved using digital PCR, a technique that enables absolute quantification of nucleic acid copy numbers. Digital PCR has been used for the diagnosis of fetal aneuploidy^[56]^. One commonly targeted gene for this purpose is *Amelogenin*, which has homologous alleles on the X (AMELX) and Y (AMELY) chromosomes. These alleles differ slightly in sequence, allowing specific probes to distinguish between them and quantify their respective copy numbers. The AMELX/AMELY ratio can then be used to accurately estimate the mLOY percentage in DNA samples^[57]^. Digital PCR can also be combined with multiplexed droplet-based single-cell platforms to quantify mLOY and mLOX in individual cells or nuclei from patient samples^[58]^. Similarly, single-cell or single-nucleus RNA sequencing can be used to assess mLOY by detecting the absence of Y chromosome gene expression^[48,59–61]^. These integrated approaches have enabled a detailed and large-scale characterization of hematopoietic sex chromosome aneuploidy.

**Figure 2.**
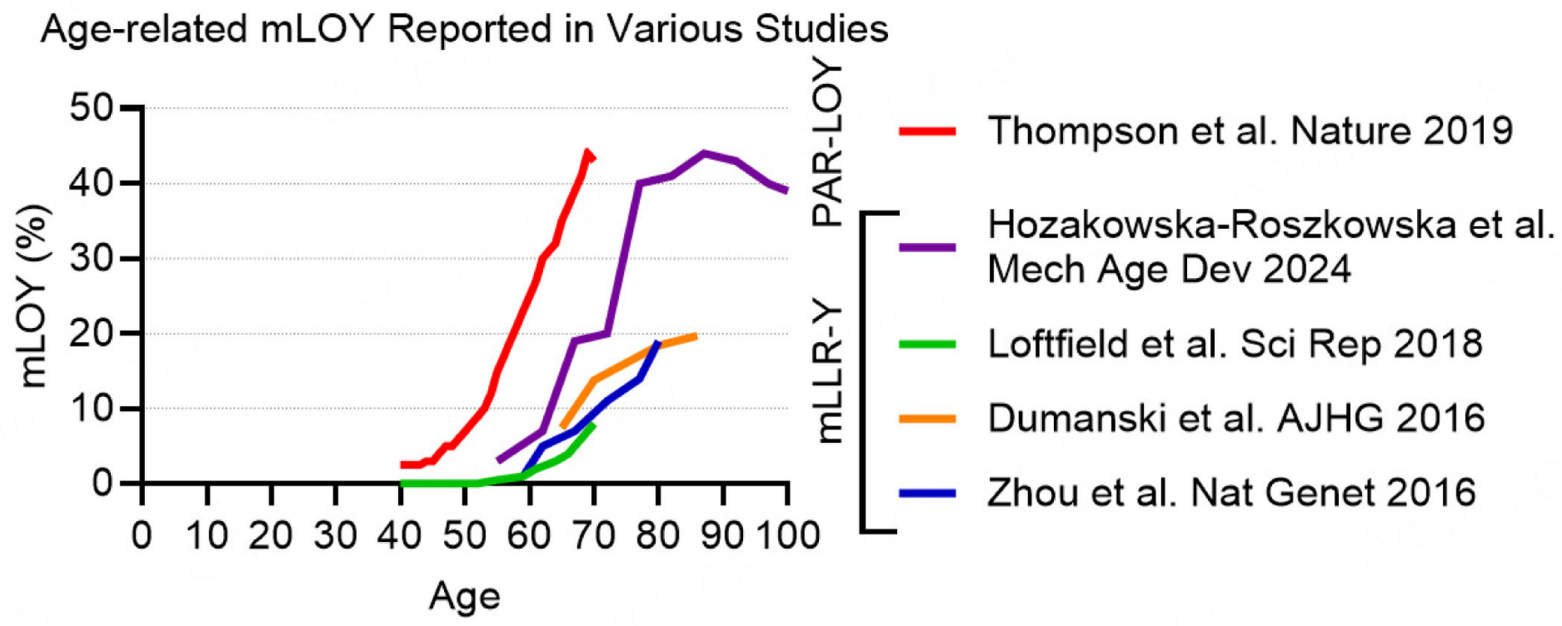
Percentage of detectable mLOY across age in different studies using mLLR-Y and PAR-LOY detection methods.

Studies involving large participant cohorts have confirmed the early observation that mLOY increases with age, while also providing more precise quantification. The mLLR-Y method was first applied to SNP data from male participants in the UK Biobank and other cohorts, suggesting that mLOY demonstrating begins to rise around age 60, and affects ~20% of men by age 80^[52–54]^. Later, the PAR-LOY method was used to analyze WGS data from the UK Biobank, which improved the sensitivity for detecting low-level mLOY. This method estimated that ~20% of all male participants exhibited detectable mLOY^[48]^. Prevalence was found to vary significantly by age: mLOY was detected in only ~2.5% of men under 45 years old, but increased linearly with age, reaching ~45% in men aged 70^[48]^. These enhanced detection capabilities have revealed the high burden of mLOY in the general population, as demonstrated by comparing mLOY percentages by age across studies using WGS [Figure 2]. Indeed, single-cell RNA sequencing of blood samples from 29 men aged 64–94 detected LOY cells in every individual^[59]^, suggesting that mLOY may occur earlier than can be detected by bulk WGS methods. Collectively, these studies confirm that age is a driving factor for mLOY prevalence in the general population.

### Drivers and risk factors for loss of Y chromosome

The drivers of Y chromosome loss are thought to resemble those of general genomic instability and subsequent mosaic chromosomal alterations in cells. Although the initial induction of LOY in a single cell remains poorly understood, it is hypothesized to result from chromosomal segregation errors during mitosis^[62]^, a common cause of broader chromosomal alterations^[63]^. The small size of the Y chromosome and its high content of repetitive sequences may render it more susceptible to complete chromosomal loss during cell division^[63,64]^. One proposed mechanism involves cellular proliferation and shares similarities with cellular senescence - a state of permanent growth arrest typically associated with aging and driven, in part, by replicative stress^[65]^. This proliferative mechanism aligns with the age-related accumulation of hematopoietic cells lacking the Y chromosome, as hematopoietic stem and progenitor cells bear a high proliferative burden and accumulate somatic mutations with age^[66]^.

In addition to proliferative mechanisms, both genetic and modifiable risk factors for mLOY have been identified. The first genetic risk locus for mLOY was discovered in the *TCL1A* gene, based on a cohort of ~13,000 individuals with and without cancer. A subsequent epigenetic analysis of UK Biobank participants estimated the heritability of mLOY at 31.7%, identifying 156 autosomal genetic loci associated with mLOY risk. Many of these loci are involved in cell cycle regulation, cancer susceptibility, and tumor growth^[48]^. These findings supported earlier studies that linked genetic variants associated with mLOY to similar biological pathways^[67]^. A polygenic risk score derived from these loci further suggested that a combined genetic approach may improve the prediction of mLOY susceptibility in men^[68]^. Notably, several of these loci were also found to influence health outcomes in women, indicating that some genetic determinants of mLOY may reflect broader mechanisms of genomic instability. A separate GWAS analysis of mLOY identified 50 genetic markers and revealed correlations with hematopoietic functions^[69]^, mirroring patterns observed in age-related myeloid skewing.

Among modifiable risk factors, smoking has emerged as the strongest and most consistent driver of mLOY^[49,54,70]^. Current smokers exhibit a threefold increase in mLOY risk, while former smokers show a return baseline (non-smoker) risk levels after cessation^[49]^. A likely underlying mechanism is increased susceptibility to DNA damage. Smoking induces DNA damage^[71]^, disrupts the hematopoietic stem cell niche^[72]^, promotes mutations during cell proliferation^[73]^, and drives tumor development through the selection of oncogenic mutations^[74,75]^. Supporting this notion, exposure to outdoor air pollution has also been linked to mLOY in older men^[76]^, suggesting that environmental toxins may contribute to mLOY through similar DNA damage pathways as smoking. DNA damage is a well-established driver of cellular aging, causing genetic aberrations and altered cell fates that contribute to tissue dysfunction^[77]^. It can also induce cellular senescence^[78]^, both of which are hallmarks of aging^[79]^. Consistently, age has been identified as the most significant risk factor for mLOY. While it remains challenging to disentangle the effects of mLOY from those of biological aging in age-related diseases, particularly cardiovascular conditions, recent large-cohort studies have begun to reveal clinical associations between mLOY and age-related pathologies that are independent of chronological age.

### Clinical associations between loss of the Y chromosome and age-related cardiovascular disease

The use of large participant cohorts combined with novel methods for mLOY detection has resulted in the identification of clinical associations with many age-related diseases. Several recent studies have concluded that mLOY is strongly correlated with increased early mortality in men^[48,50,80]^. Moreover, mortality risk rises in proportion to the fraction of leukocytes exhibiting LOY^[81]^. Reported hazard ratios of 2.0 or higher suggest that elderly men with mLOY may live only half as long as their counterparts without mLOY^[80,82]^. This elevated mortality is mainly explained by an increased prevalence of age-related diseases. Men with hematopoietic mLOY face a higher risk of cancer incidence and mortality^[50,54,83,84]^, Alzheimer’s disease^[52,85,86]^, age-related lung disease^[70]^, and mortality from chronic kidney disease^[87]^, which are all common age-associated diseases. Additionally, mLOY has been linked to age-related macular degeneration^[88,89]^, autoimmune thyroiditis^[90]^, diabetes^[53]^, and MDS-related myeloid neoplasia^[91]^.

Recent studies have further detailed the relationship between mLOY and cardiovascular diseases, indicating that the risk may be elevated for specific cardiovascular conditions [Table 1]. The first reported association between mLOY and cardiovascular health emerged in 2017, in a study of 366 men undergoing carotid endarterectomy. mLOY was significantly correlated with larger atheroma size and increased incidence of secondary major cardiovascular events, although not with baseline severity of cardiovascular disease^[92]^. Notably, mLOY was also detected within atherosclerotic plaques, suggesting potential direct functional roles for mLOY in cardiovascular pathology^[93]^. A larger analysis of the UK Biobank found that individuals with mLOY (defined here as > 40% of leukocytes exhibiting LOY) were at increased risk of mortality from several circulatory system diseases, including hypertensive heart disease, heart failure, congestive heart failure, and aortic aneurysm or dissection^[81]^. A smaller, independent study also linked mLOY to abdominal aortic aneurysms^[47]^. Interestingly, atherosclerosis and coronary artery disease (CAD) were not significantly associated with mLOY, which aligns with earlier findings that mLOY correlated with secondary major cardiovascular events rather than baseline atherosclerotic burden^[92]^. Separately, somatic mosaic chromosomal alterations have been associated with CAD in cancer survivors, but mLOY alone was not a significant contributor^[40]^. mLOY has also been linked to an increased risk of hospitalization for atrial fibrillation^[55]^. Collectively, these findings suggest that mLOY may predict future cardiovascular mortality through chronic effects on tissue function, rather than through acute inflammation-related events. Supporting this, mLOY has been associated with worse outcomes on the modified Rankin Scale following stroke^[94]^, indicating a potential impact on tissue function that is independent of inflammatory processes.

As both mLOY and CHIP mutations increase with age and are detectable through sequencing, and as they share similar risk factors, it is notable that they appear to follow distinct pathological mechanisms. Comparisons between CHIP and mLOY, as discussed in this review, are summarized in Table 2. The age-related rise in mLOY prevalence and its correlation with fibrotic (rather than inflammatory) cardiovascular conditions underscore the urgent need to elucidate the causal mechanisms by which mLOY contributes to age-related cardiovascular disease.

### Causal studies and molecular consequences of loss of the Y chromosome and cardiovascular disease

Murine models of hematopoietic mLOY were generated to assess its causal role in cardiovascular pathology and to determine whether mLOY directly contributes to disease or merely reflects aging. Hematopoietic mLOY was induced in mice through CRISPR/Cas9-mediated deletion of the Y chromosome in hematopoietic stem cells by inserting gRNAs targeting repetitive Y chromosome sequences, followed by bone marrow transplantation into male recipient mice. These mLOY mice exhibited reduced lifespan and age-associated cognitive decline^[81]^, mirroring clinical observations. Compared to controls, aged mLOY mice developed more fibrotic deposits in the heart, lungs, and kidneys, and showed a decline in cardiac function, suggesting accelerated age-related cardiomyopathy^[81]^. Additionally, mLOY impaired myocardial remodeling after cardiac injury. In the transverse aortic constriction pressure-overload model, mLOY mice exhibited reduced fractional shortening, increased fibrosis, and expansion of fibroblasts^[81]^. Mechanistically, monocytes with mLOY recruited to injured hearts preferentially polarized into pro-fibrotic rather than pro-inflammatory macrophages, leading to elevated levels of TGF-β1 in cardiac tissue. Treatment with TGF-β1-blocking antibodies attenuated the fibrotic response and improved cardiac output^[81]^. This shift in macrophage polarization may help explain why mLOY is associated with fibrotic, but not inflammatory, cardiovascular diseases in clinical studies.

The consequences of mLOY on gene expression and cellular function are widespread, with broad implications for cardiovascular disease. Clinically, developmental X chromosome loss (X0), either globally or with high mosaicism, leads to Turner Syndrome - characterized by short stature, gonadal dysgenesis, dysmorphic features, and organ abnormalities - and is associated with a high prevalence of congenital heart defects, bicuspid aortic valve, coarctation of the aorta, hypertension, and increased cardiovascular mortality^[95]^. In adult men, mLOY in leukocytes alters the expression of over 500 autosomal genes^[59]^. Similar gene expression disruptions have been observed in cardiac leukocytes from patients with dilated cardiomyopathy^[61]^, suggesting that mLOY exerts pleiotropic effects that vary by cell type and tissue. These effects may arise from the epigenetic regulatory roles of specific MSY genes expressed in leukocytes, including the histone demethylases *UTY*^[96]^ and *KDM5D*^[97]^, and the RNA helicase *DDX3Y*^[98]^. Deletion of these Y-linked genes appears to influence autosomal gene expression and cellular function, suggesting that certain Y chromosome genes have non-reproductive roles relevant to aging-related diseases.

To explore the specific role of Y chromosome genes in cardiovascular disease, murine models were developed using XY* mice, which allow selective deletion of Y-linked genes while preserving Y centromere function^[99]^. Hematopoietic stem cells from XY* mice were transplanted into recipient mice, and the recipients displayed the same pro-fibrotic cardiac response and impaired functional recovery after heart injury^[61]^. A CRIPSR screen identified *Uty* as the sole Y-linked gene whose deletion in hematopoietic stem cells reproduced the fibrotic cardiac phenotype observed in mLOY mice^[61]^. Single-cell chromatin accessibility analysis of cardiac leukocytes lacking *Uty* revealed widespread changes in chromatin structure, including increased accessibility near pro-fibrotic macrophage gene loci in circulating monocytes^[61]^. These findings indicate a key role for *UTY* in mediating mLOY-associated cardiac fibrosis. However, additional Y chromosome genes may also influence age-related outcomes. Some Y-linked genes have oncogenic potential, as shown in cancer-related studies^[100,101]^. Regulatory T cells show high mLOY burdens, which may influence cancer susceptibility through alternative mechanisms^[102]^. Moreover, mLOY leukocytes showed reduced *CD99* expression, impairing transendothelial migration and immune cell homing^[60]^. Therefore, although *Uty* deletion recapitulates the cardiac phenotype of mLOY, other Y-linked genes likely contribute to the broader spectrum of age-related diseases and early mortality associated with mLOY.

These mechanistic studies have been instrumental in establishing mLOY as a driver of age-related pathology. As genomic instability - a hallmark of aging - is both a cause and consequence of mLOY, it was crucial to isolate the direct effects of mLOY on leukocyte function and disease. The epigenetic regulatory role of UTY suggests a mechanistic link between mLOY and biological aging. As described above, epigenetic clocks use methylation patterns in leukocytes to estimate biological age and correlate with cardiovascular disease risk. Given UTY’s influence on epigenetic modifications, mLOY may directly affect leukocyte aging. Conversely, epigenetic dysregulation could also promote mLOY, as disrupted chromatin regulation is a known driver of mutations and chromosomal alterations in genomic instability^[103]^. Additionally, genes that predispose individuals to mLOY are regulated by chromatin accessibility in hematopoietic cells^[48]^, supporting a feedback loop between epigenetic regulation and mLOY induction. In conclusion, these experimental studies demonstrate that mLOY contributes independently to cardiovascular pathology, beyond its association with chronological aging. Further studies are necessary to disentangle the complex relationships among mLOY, epigenetic regulation, and leukocyte aging. These findings highlight the exciting potential of mLOY as a novel therapeutic target for age-related disease in elderly men.

## FUTURE DIRECTIONS

Recent findings suggest that mLOY may contribute to age-related pathologies through multiple mechanisms, highlighting its potential as an underlying driver of aging in men. Numerous promising avenues for future research aim to elucidate the overall impact of mLOY, especially in the context of aging.

The functional consequences of mLOY in macrophages, namely enhanced pro-fibrotic signaling and reduced pro-inflammatory signaling, contrast with those observed in CHIP. CHIP-associated mutations promote pro-inflammatory responses in macrophages and other leukocytes, contributing broadly to chronic inflammation in age-related diseases^[23,24]^. Both correlative and mechanistic studies have implicated CHIP in the development of heart failure^[27]^. In contrast, mechanistic and clinical studies of mLOY suggest a pro-fibrotic, potentially anti-inflammatory effect on leukocytes. Specifically, while both mLOY and CHIP-associated mutations induce clonal expansion of hematopoietic stem cells and affect macrophage behavior in cardiac tissue, their downstream effects differ. mLOY enhances macrophage-mediated TGFβ signaling, directly promoting fibrosis, whereas CHIP mutations (e.g., in TET2) increase macrophage-mediated IL1β and NLRP3 inflammasome activity, which contributes to cardiac remodeling and reactive fibrosis^[104]^. Although these mechanisms diverge, both somatic mutations are age-related and drive cardiovascular mortality, suggesting potential interplay between CHIP and mLOY during aging. Several studies have hypothesized a connection between CHIP mutations and mLOY, with emerging evidence suggesting they may act in concert to promote disease^[105–107]^. However, more detailed mechanistic studies are required to disentangle their distinct and potentially opposing effects.

A major limitation in studying mLOY mechanisms is the scarcity of appropriate experimental models. Current murine models rely on bone marrow transplantation following lethal irradiation to introduce hematopoietic stem cells lacking the Y chromosome into recipient mice. Although this approach has been instrumental in uncovering mLOY-related mechanisms, it models hematopoietic chimerism rather than mosaicism. More accurate preclinical models that avoid transplantation are needed to better capture hematopoietic mosaicism and investigate the relationship between mLOY and cardiovascular disease progression. The development of such models will significantly advance mechanistic insights and enable discovery of causal disease links.

Recent studies have also revealed a higher-than-expected prevalence of mLOX in women^[51,108]^. Analyses across several cohorts have identified mosaic chromosomal alterations affecting the X chromosome in ~12% of women in the general population, with associations to age, smoking, and leukemia, and with identified genetic determinants of mLOX^[51]^. However, mLOX is more challenging to quantify and investigate than mLOY because mLOX results in haploinsufficiency of the X chromosome, whereas mLOY involves complete loss of the Y chromosome. Future studies capable of overcoming these challenges will be essential for determining the similarities and differences between mLOX and mLOY. Interestingly, both mLOY and mLOX generate X0 genotype immune cells, suggesting potential common mechanisms contributing to age-related pathology. The loss of one X chromosome in mLOX may be functionally analogous to mLOY in terms of haploinsufficiency of X-Y homologous genes that escape X-chromosome inactivation. These X0 immune cells may have distinct impacts on cardiovascular disease progression in men and women. Notably, a study by Lim *et al.* that investigated the association between mLOY and atrial fibrillation also found that mLOX was inversely associated with atrial fibrillation (HR = 0.9), suggesting a potential protective effect^[55]^. In contrast, a separate study using UK Biobank data found that mLOX was uniquely predictive of lymphoid leukemia risk (HR = 2.5)^[38]^. These findings underscore the need for future research to compare sex-specific differences and phenotypic outcomes of mLOY and mLOX in aging and disease.

The translational implications of these recent correlative and causal findings on mLOY in age-related cardiovascular disease are highly promising. Screening elderly populations for hematopoietic clonal expansions, including assessing mLOY in older men, could provide valuable information for risk stratification. mLOY can be detected using several methods described in this review, including WGS/WES for genetic risk profiling, or ddPCR when blood samples are available. However, it is currently not feasible to assign precise disease risk to individuals based on mLOY percentage. Further research is needed to define mLOY thresholds associated with increased risk for specific cardiovascular diseases. It is likely that some diseases are more susceptible to hematopoietic alterations from mLOY than others. Additionally, future studies should aim to identify reliable biomarkers for mLOY to enhance age-associated cardiovascular risk assessment. Ultimately, research should focus on developing and testing therapeutic strategies to restore hematopoietic function disrupted by mLOY. Although TGFβ inhibition has shown promise in preclinical models, targeting this pathway carries substantial risk for off-target effects. Potential therapeutic approaches may be informed by clinical advances in CHIP-related interventions. For instance, the CANTOS clinical trial found that the IL-1β inhibitor Canakinumab significantly reduced major adverse cardiovascular events in post-myocardial infarction patients with TET2 CHIP mutations^[109]^. This suggests that clonal hematopoietic mutations may inform future clinical decision making. Nevertheless, as with CHIP^[110]^, deeper mechanistic understanding is essential before mLOY-directed therapies can be realized.

## CONCLUSIONS

Mosaic sex chromosome aneuploidy is an emerging topic that seems to be intrinsically linked to biological aging and may play a causal role in age-related pathologies, particularly those involving cardiovascular disease. Its high prevalence in the general population and strong correlations with mortality and age-related diseases have shifted the traditional view of sex chromosome function beyond reproductive roles. The Y chromosome should no longer be viewed as a genetic wasteland. Causal studies have demonstrated that the *UTY* gene on the Y chromosome promotes pro-fibrotic gene expression in macrophages through epigenetic regulation. Loss of this function in mLOY directly contributes to cardiac fibrosis via TGFβ signaling (see graphical abstract). Hematopoietic mLOY is now associated with several hallmarks of aging, including genomic instability, epigenetic alterations, disrupted intercellular communication, and chronic inflammation. Future studies may uncover additional links to other aging-related processes. Investigating how mosaic sex chromosome aneuploidy drives biological aging could reveal novel mechanisms and potential therapeutic targets for age-associated diseases.

## Figures and Tables

**Table 1.** Summary of clinical studies on cardiovascular conditions associated with mLOY

Publication	Study population	Detection method	mLOY-associated cardiovascular conditions	Odds/hazard ratio

Haitjema *et al*., 2017^[92]^	366 carotid endarterectomy patients (Athero-Express Biobank; 3-year follow-up)	mLLR-Y	Atheroma size (> 10%) Secondary cardiovascular endpoints	OR = 2.15 HR = 2.28
Tang *et al*., 2019^[47]^	37 AAA, 12 AOD, 91 healthy controls (China Medical University Aneurysm Biobank)	FISH	Abdominal aortic aneurism	OR = 1.64
Sano *et al*., 2022^[81]^	223,550 participants (UK Biobank; 11.5-year median follow-up)	PAR-LOY	Mortality from circulatory system diseases Hypertensive heart disease Heart failure Congestive heart failure Aortic aneurysm and dissection	HR = 1.31 HR = 3.48 HR = 1.76 HR = 2.42 HR = 2.76
Lim *et al*., 2024^[55]^	190,613 participants (UK Biobank)	mCA caller	Atrial fibrillation	HR = 1.06

**Table 2.** Comparison of factors between CHIP mutations and mLOY

	Clonal hematopoiesis of indeterminate potential mutations			Hematopoietic mosaic loss of Y chromosome		

Prevalence rates	Age (all genders) 40 years 60 years 80 years	Prevalence 0%−1% 2%−5% 8%−12%		Age (males) 40 years 60 years 80 years	Prevalence 0%−2% 5%−25% 20%−45%	
Detection methodologies	Method	Relative sensitivity	Relative cost	Method	Relative sensitivity	Relative cost
	WGS/WES Targeted Seq Error-corrected scRNA-seq	+ +++ ++++ +++++	++ + +++ +++++	mLLR-Y PAR-LOY ddPCR scRNAseq	+ ++ +++ +++++	++ ++ + +++++
Risk factors	Age; smoking; environmental toxins; genetic predisposition; obesity; chemotherapy			Age; smoking; environmental toxins; genetic predisposition		
Molecular signatures	Recurrent point mutations in genes (DNMT3A, TET2, ASXL1, PPM1D, JAK2; accounting for ~90% of cases)			Mosaic chromosomal abnormality in the Y chromosome		
Associated cardiovascular diseases	• Ischemic heart failure • Atherosclerosis • Venous thrombosis			• Congestive heart failure • Aortic aneurysm and dissection • Atrial fibrillation		

+ : Lower;

+++++ : higher.
